# Genetic mapping of QTLs controlling brown seed coat traits by genome resequencing in sesame (*Sesamum indicum* L.)

**DOI:** 10.3389/fpls.2023.1131975

**Published:** 2023-02-23

**Authors:** Han Wang, Chengqi Cui, Yanyang Liu, Yongzhan Zheng, Yiqing Zhao, Xiaoqin Chen, Xueqi Wang, Bing Jing, Hongxian Mei, Zhonghua Wang

**Affiliations:** ^1^ State Key Laboratory of Crop Stress Biology for Arid Areas, College of Agronomy, Northwest A&F University, Yangling, China; ^2^ Henan Sesame Research Center, Henan Academy of Agricultural Sciences, Zhengzhou, China; ^3^ The Shennong Laboratory, Zhengzhou, China

**Keywords:** sesame, seed coat color, whole-genome resequencing, BSA, QTL mapping, qRT-PCR

## Abstract

**Introduction:**

Sesame seeds have become an irreplaceable source of edible oils and food products with rich nutrients and a unique flavor, and their metabolite contents and physiological functions vary widely across different seed coat colors. Although the quantitative trait loci (QTLs) for genetic variation in seed coat color have been extensively investigated, the identification of unique genetic loci for intermediate colors such as brown has not been reported due to their complexity.

**Methods:**

Here, we crossed the white sesame ‘Yuzhi No. 8’ (YZ8) and the brown sesame ‘Yanzhou Erhongpi’ (YZEHP) to construct a recombinant inbred line (RIL) population with consecutive self-fertilization for ten generations.

**Results:**

The selfed F1 seeds were brown which was controlled by a dominant gene. Based on the genotyping by whole-genome resequencing of the RILs, a major-effect QTL for brown coat color was identified through both bulk segregant analysis (BSA) and genetic linkage mapping in sesame, which was located within a 1.19 Mb interval on chromosome 6 (qBSCchr6). Moreover, we found that the YZEHP seed coat initially became pigmented at 20 days post-anthesis (DPA) and was substantially colored at 30 DPA. We screened 13 possible candidate genes based on the effects of genetic variants on protein coding and predicted gene functions. Furthermore, qRT‒PCR was used to verify the expression patterns of these genes in different post-anthesis developmental periods. We noted that in comparison to YZ8 seeds, YZEHP seeds had expression of SIN_1023239 that was significantly up-regulated 2.5-, 9.41-, 6.0-, and 5.9-fold at 15, 20, 25, and 30 DPA, respectively, which was consistent with the pattern of brown seed coat pigment accumulation.

**Discussion:**

This study identified the first major-effect QTL for the control of the brown seed coat trait in sesame. This finding lays the foundation for further fine mapping and cloning as well as investigating the regulatory mechanism of seed coat color in sesame.

## 1 Introduction

Sesame (*Sesamum indicum* L.) is an exceptional and essential oilseed crop; it is one of the oldest such crops known to mankind, having been domesticated from its wild progenitor *S. malabaricum* on the Indian subcontinent approximately 5000 years ago (Bedigian, 2003; Fuller, 2003). Sesame seeds are used for a wide variety of applications, both as condiments and as a source of edible oil. Sesame oil is commonly used for its distinctive flavor, in addition to being a key component in the production of margarine, soap, and lubricants (Hwang, 2005). One of the main distinguishing characteristics of sesame seeds is the color of the seed coat. Seed coat color is a crucial aspect of seed quality and is related to the biochemical properties of the seed and to the activity and content of its antioxidant substances (Shahidi et al., 2006; Kermani et al., 2019). These different biochemical and antioxidant properties may be most closely related to higher levels of sesamol, sesaminol, alpha-tocopherol, and flavonoids in the seed coats of colored sesame than that of white sesame seeds (Xu et al., 2005). However, it has not yet been possible to identify the genes that regulate the metabolic pathways and mechanisms of interaction that determine sesame seed coat color, which is typically thought to show a complicated pattern of quantitative inheritance (Zhang et al., 2013).

Mature sesame seeds come in a variety of natural coat colors, including black, gray, brown, gold, yellow, beige, and white (Prasad and Gangopadhyay, 2011; Pandey et al., 2013). As seed coat color is one of the central targets of sesame breeding programs, research into the inheritance of the trait and the corresponding gene loci have been of considerable scientific interest. In 1931, a Japanese researcher initially suggested that the inheritance of sesame seed coat color potentially fit a segregation pattern involving three allelic genes (Teshima, 1931). Zhang et al. (2013) identified and analyzed the genetic segregation of quantitative trait loci (QTLs) for sesame seed coat color over six generations and concluded that two major-effect genes with additive-dominant-epistatic effects and multiple minor-effect genes with additive-dominant-epistatic effects were responsible for controlling the seed coat color trait. Moreover, seven QTLs that control sesame seed coat color traits were identified by Du et al. (2019). In addition, Wang et al. (2016) mapped three QTLs that were repeatedly detected and accounted for 80% of the phenotype variation by resequencing a RIL population. According to the annotation of genes anchored to genomic intervals combined with transcriptome analysis, the polyphenol oxidase (PPO) gene may be involved in the production of the black seed coat in sesame, and this finding has been supported by several investigations (Wei et al., 2015; Wang et al., 2016; Wei et al., 2016; Wang et al., 2020). Furthermore, since the development of next-generation sequencing technologies, whole-genome association analysis has been used to dissect complex traits in crops, as QTL mapping research in the segregating progeny of classical hybrids is limited by a low number of recombination events and cultivar-specific allelic loci (Nordnorg and Welgel, 2008; Guo et al., 2013). By resequencing an association analysis panel of 366 sesame germplasm lines, Cui et al. (2021) demonstrated complex genetic variation in seed coat color. The results revealed that 22 significant single-nucleotide polymorphisms (SNPs) were located within the reported QTL confidence intervals and that the four most reliable and significant flanking regions of these SNPs contained 92 candidate genes. However, researchers have been unable to perform additional in-depth investigations on the locus that controls the seed coat color trait in sesame due to gaps in the QTL mapping studies regarding intermediate seed coat colors. Furthermore, it is not possible to validate the currently available genetic loci against each other because much of the existing sesame QTL mapping research has been based on independent genetic maps. Thus, to meet the needs of molecular breeding, QTL mapping research on sesame seed coat color should be expanded using high-quality genomes anchored to chromosomes.

Plant seed color is mainly characterized by the accumulation of pigmented metabolites in the seed coat. In this context, a brown seed coat has been identified as possibly being regulated by the flavonoid synthesis pathway in several plant species. The genes that may regulate the brown seed coat in *Arabidopsis* include those encoding the Transparent Testa12 (TT12) and EXO70 exocyst subunit (EXO70B1) transporter proteins and the proanthocyanidin (PA) oxidase enzyme (TT10) (Debeaujon et al., 2001; Pourcel et al., 2005; Kulich et al., 2013). Moreover, Transparent Testa Glabra2 (TTG2) was found to interact with TTG1 to form a complex that directly regulates the expression of TT12 to produce brown *Arabidopsis* seed coats (Gonzalez et al., 2016). Among other crops, many transcription factors, such as MYB, basic helix-loop-helix (bHLH), and WD40 proteins, have been identified as potentially being involved in the regulation of flavonoid biosynthesis (Zhang et al., 2009; Gillman et al., 2011; Hong et al., 2017; Ren et al., 2017). Small interfering RNAs (siRNAs) were also found to silence the expression of transposable elements (TEs) or protein-coding genes and thereby affect the synthesis and regulation of flavonoid metabolites (Jia et al., 2020). In addition, PPOs such as laccase, tyrosinase, and even peroxidase are involved in the oxidation steps of PA, lignin, and melanin biosynthesis (Pourcel et al., 2007; Yu, 2013).

In this study, we used a RIL population and the whole-genome resequencing technique to perform QTL mapping for seed coat color in sesame. A major-effect QTL, *qBSCchr6*, controlling the brown seed coat trait in sesame was revealed by the combination of BSA and high-density genetic linkage mapping. The candidate genes involved in the regulation of the brown seed coat were screened based on the evaluation of the effect of genetic variants on protein coding and predicted gene functions. The expression patterns of these genes in different developmental periods at post-anthesis were analyzed using qRT−PCR. The results of this study will enhance the development of research on the genetic and molecular mechanisms of sesame seed coat color regulation and provide a basis for functional gene cloning studies.

## 2 Materials and methods

### 2.1 Plant materials

The cultivar Yanzhou Erhongpi (YZEHP) has a brown seed coat and is a landrace collected from Shandong Province, China. The Yuzhi No. 8 (YZ8) cultivar, which was bred by Henan Academy of Agricultural Science, produces seeds with a white coat. A mapping population of 315 recombinant inbred lines (RILs, F_10_ generation) was constructed from a cross between YZEHP and YZ8 using the single-seed descent (SSD) method. The lines showed obvious differences in traits such as plant height, thousand grain weight, capsule length, and seed coat color. The RIL population and both parents were planted in 2020 at experimental sites in Sanya, Hainan Province (SY, N18°140′, E109°290′), Zhumadian, Henan Province (ZMD, N32°59′, E114°42′), and Nanyang, Henan Province (NY, N32°54′, E112°24′). All the plants were arranged in a randomized block design with two replicates, and 10 representative plants of each line were harvested for the investigation of seed coat color.

### 2.2 Seed coat color evaluation and statistical analysis

Initially, we superficially observed both brown and white mature seed coat types. Additionally, a Colorflex EZ spectrophotometer (Hunter Associates Laboratory Inc, Virginia, USA) was used to measure the colors of the seed coats in three different environments. Mature seeds were scanned in a quartz box to quantify the L*, a*, and b* values for seed coat color. The L* value, which represents brightness, ranges from 0 (black) to 100 (white), while the values of a* and b*, which represent color shades, range from -60 for green to +60 for red and -60 for blue to +60 for yellow, respectively (Aruldass et al., 2014). Phenotypic statistics were calculated using SAS v9.1 (SAS Institute, Inc., Cary, NC, USA). Based on the mean values of L*, a*, and b* for the sesame seed coat color phenotype among replicates and different environments, the broad-sense heritability was calculated using the AOV module in QTL IciMapping v4.2 (Meng et al., 2015). Furthermore, the color phenotypes observed for each line corresponded to the L*, a*, and b* values and were visualized by ggplot2 v3.3.6 (Wickham, 2016).

### 2.3 Sequencing and SNP/InDel calling

Genomic DNA was extracted from seedling leaves of the parents and RILs using a modified cetyltrimethylammonium bromide (CTAB) method (Mei et al., 2017). The quality of the genomic DNA was examined with a NanoDrop 2000 (Thermo Fisher Scientific, Waltham, MA, USA) and by 1.0% agarose gel electrophoresis. After ultrasound fracturing, the DNA was sequentially end repaired, sequencing junction ligated, and enriched by magnetic bead adsorption to obtain fragments with a genomic length of approximately 400 bp. These fragments were then amplified by PCR to establish a sequencing library. The Illumina NovaSeq 6000 platform was used to sequence the quality-checked libraries with a total sequencing read length of 300 bp using the Illumina PE150 sequencing strategy. The two parents and the RILs were sequenced at depths of approximately 15× and 5×, respectively. The reads were filtered to eliminate adapters and low-quality reads. Based on the seed coat color phenotypes of the RILs grown in ZMD, we merged the clean reads of 50 randomly selected lines from white and brown sesame, respectively, to construct extreme bulks. The clean reads of all samples were aligned to the reference genome (Wang et al., 2016) using Burrows–Wheeler Aligner (BWA) v0.7.17 (Li and Durbin, 2009). SNPEff v4.3T (Cingolani et al., 2012) and the gene annotation information of the reference genome were used to functionally annotate SNPs and small InDels after correction and detection by using Genome Analysis Toolkit (GATK) v4.0.11.0 (McKenna et al., 2010) and SAMtools v1.9.0 (Li et al., 2009). According to genetic principles, all markers were examined for parental polymorphism. Variant sites that differed between the parents were selected and coded as molecular markers, and the genotypes of the RILs and bulks were extracted for additional analysis.

### 2.4 BSA, genetic map construction, and QTL mapping

The QTL-seq method was implemented to calculate the ΔSNP index (Takagi et al., 2013). The SNP index represents the proportion of short reads harboring SNPs that differ from the reference sequence to the total reads covering a particular genomic position (Abe et al., 2012). The SNP index of the extreme bulks was statistically analyzed based on the average SNP index within each genomic interval containing 20 SNP variants, which was individually measured using a sliding window of 5 SNP variants. The ΔSNP index is the average SNP index difference between the two extreme bulks (99.9% quantile as the threshold), and this analysis revealed significant differences in genotype frequencies between the extreme bulks (Hill et al., 2013).

We selected polymorphic markers of the aa×bb type between the parents as valid markers, and these markers were screened for abnormal bases, completeness, and segregation distortion after being used to genotype the RIL population. Moreover, we utilized a reference genome assisted correction-based linkage group ordering scheme. We completed the construction of the genetic map using MstMap (Wu et al., 2008), and we then used ASMapR v1.0-4 and R/qtl v1.44-9 to evaluate the monomeric origin and recombination relationships (Broman et al., 2003; Taylor and Butler, 2017). In addition, we analyzed the collinearity of the linkage map with the physical map. Finally, the visualization of the genetic map was completed using LinkageMapViewR v2.1.2 (Ouellette et al., 2018). R/qtl was used for standard and stepwise interval mapping with 1000 permutations and a *p* value of 0.05 as the logarithm of odds (LOD) significance detection threshold. Composite interval mapping (CIM) was performed based on a 5 cM marker window size and a step of 1 cM. The location of each QTL was determined based on the location of the LOD peak for each QTL and the surrounding area. The percentage of phenotypic variation explained (*R^2^*) by the QTL was estimated at the highest probability peak (Tao et al., 2022).

### 2.5 Bioinformatic analysis

Gene sequence information was obtained based on the candidate intervals. The functions of the candidate genes were annotated by using the NR (http://www.ncbi.nlm.nih.gov/), UniProt (http://www.uniprot.org/), Gene Ontology (GO) (http://www.geneontology.org/), Kyoto Encyclopedia of Genes and Genomes (KEGG) (http://www.genome.jp/kegg/) databases, and the Basic Local Alignment Search Tool (BLAST) program in the EggNOG (http://eggnog-mapper.embl.de/) database for annotation. Moreover, the analysis of protein coding variants included variants annotated by SNPEff with sequence ontology terms for assessing sequence changes and impacts, and categorized the impact of SNP/InDel within the candidate interval into four classes: High, Moderate, Low, and Modifier, in descending order according to the effect of the variant on protein coding (**Supplementary Table 1**) (Cingolani et al., 2012; Oren et al., 2022).

### 2.6 RNA extraction and qRT−PCR analysis of candidate genes

We also sampled parental seeds at 10, 15, 20, 25, and 30 DPA in Yangling, Shaanxi Province (N34°27′, E108°07′), in 2022. Quantitative color analysis of the seed coat was performed with a CIE-Lab color scale (Colorimeter, CS-820, Hangzhou, China) with a 6 mm aperture due to the small sample size (Dong et al., 2022). All samples were flash frozen in liquid nitrogen and stored at -80°C in the refrigerator until needed. Total seed RNA was extracted using a kit (DP441, TIANGEN, China) and first-strand cDNA was synthesized by the PrimeScript RT reagent kit (#6210A, Takara, Kusatsu, Japan). Three independent biological replicates of the qRT−PCR (#RR820A, Takara, Kusatsu, Japan) protocol were tested using cDNA as the template for each experiment. The sesame actin gene (SIN_1006268) was used as the internal reference gene (Wei et al., 2015), and relative gene expression was calculated using the 2^− ΔΔCT^ method (Livak and Schmittgen, 2001).

## 3 Results

### 3.1 Phenotypic and genetic analysis of the brown seed coat in sesame

**Figure 1:**
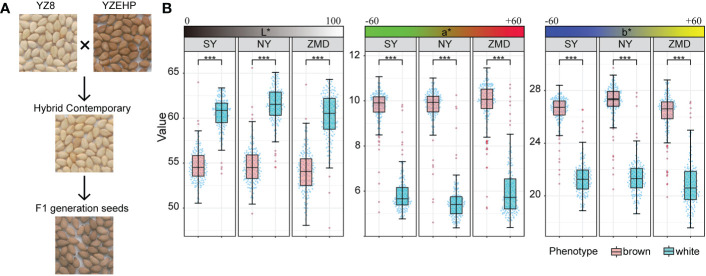
Phenotypic analysis of parents and RILs. **(A)** Seed coat color of the parents and hybrid offspring. **(B)** Distribution of quantitative values of L*, a*, and b* in the RIL population, Significant levels were determined by T-test, with *** representing *p*<0.001 level.

To reveal the genetic basis of the brown seed coat color in sesame, a RIL population including 315 lines was developed using YZEHP (male, brown seeds) and YZ8 (female, white seeds) as two parental lines in this study. We first investigated the phenotypes of seed coat color traits for several generations. The contemporary hybrid seeds obtained from the maternal plants were white (consistent with the YZ8 phenotype), while the selfed seeds developed from the F_1_ generation were brown (consistent with YZEHP phenotype) (**Figure 1A**). The brown seed coat in sesame is dominant to the white seed coat. Notably, angiosperm seed coats develop from bead tepals (Haughn and Chaudhury, 2005). Therefore, the genotype of the sesame seed coat is consistent with that of the female parent because the inheritance of sesame seed coat traits is matrilineal, as found in previous studies (Wang et al., 2016; Das et al., 2018). We performed visual observations of mature seed color phenotypes and identified 162 and 153 lines among 315 RILs with brown and white seed coats, respectively (data not shown). Furthermore, we quantified the seed coat color by using a colorimeter and found that the L*, a*, and b* values of the brown and white seeds of the RILs differed significantly (*P*<0.001) across the three environments (**Figure 1B**). Interestingly, the L*, a*, and b* values showed a bimodal continuous distribution in the RIL population (**Supplementary Figure 1**). Additionally, the mean coefficients of variation (CV) for the L*, a*, and b* values across environments were 6.54%, 27.86%, and 12.80%, respectively. The L* value for RILs across environments ranged from 49.23~64.63, the a* value ranged from 4.51~11.18, and the b* value ranged from 18.36~28.97. The L*, a*, and b* values presented average broad-sense heritabilities of 94.95%, 96.87%, and 95.67%, respectively (**Figure 1B**; **Table 1**). The results suggest that the phenotype of the brown seed coat trait in sesame is determined (in order from highest to lowest) by redness, yellowness, and brightness.

**Table 1:** Descriptive statistics and broad-sense heritability (*H* ^2^) for three seed coat color related traits of RILs.

Trait	Environment	Mean	SD	Range	CV (%)	Excess Kurtosis	Skewness	*H* ^2^ (%)
L*	SY	57.51	3.41	50.54-63.99	5.94	-1.33	0.04	94.95
	NY	58.01	3.95	49.36-65.59	6.82	-1.32	0.06	
	ZMD	57.01	3.92	47.79-64.3	6.88	-1.04	0.01	
	Mean	57.51	3.76	49.23-64.63	6.54	-1.23	0.04	
a*	SY	7.86	2.09	4.77-11.07	26.55	-1.76	-0.07	96.87
	NY	7.67	2.28	4.37-11.01	29.78	-1.81	-0.04	
	ZMD	8.04	2.21	4.39-11.46	27.49	-1.65	-0.14	
	Mean	7.86	2.19	4.51-11.18	27.86	-1.74	-0.08	
b*	SY	23.99	2.85	18.87-28.40	11.87	-1.63	-0.12	95.67
	NY	24.34	3.20	18.64-29.72	13.15	-1.65	-0.09	
	ZMD	23.64	3.16	17.57-28.80	13.35	-1.49	-0.14	
	Mean	23.99	3.07	18.36-28.97	12.80	-1.59	-0.12	

SD, standard deviation; CV, coefficient of variation; *H* ^2^ broad-sense heritability.

### 3.2 Sequencing the RIL population for BSA analysis and marker identification

Whole-genome resequencing was used to analyze the two parents and 315 RILs. A total of 455.90 Gb of clean bases was obtained after sequencing and filtering; the average Q30 quality score was over 90.98%; the average matching efficiency of the samples to reference genome was 97.18%; and the GC content ranged from 36.67~39.3%. The amounts of data obtained for YZEHP and YZ8 were 5.24 Gb and 4.93 Gb, respectively, and the actual average amount of data obtained for the RILs was 1.41 Gb, and the average sequencing coverage was 18.61× for the parents and 5.16× for the RIL population (**Supplementary Figure 2**; **Supplementary Table 2**). It was evident that all samples showed a sufficient amount of data, normal distribution, and regular sequencing results when compared to the sesame reference genome, suggesting that they could be used for subsequent analysis. Then, we merged the clean reads separately from 50 lines to develop the following two extreme bulks: one with 231 million reads in a white seed coat bulk and the other with 240 million reads in a brown seed coat bulk (**Supplementary Table 2**). These two extreme bulks were screened for 38,752 SNP markers, which were used to calculate genotype frequencies (**Supplementary Table 3**). Additionally, 1,284,658 SNP/InDel markers were detected between two parental lines, of which 167,862 were valid markers of the aa×bb type with a sequencing depth of no less than 2 in the RILs and 10 in the parental lines (**Supplementary Figure 3**). After screening the markers for abnormal bases, completeness, and segregation distortion, 7,908 high-quality markers remained after genotyping the RIL population with validated polymorphic markers were used for the following analysis.

### 3.3 Construction of a high-density genetic map

Among the remaining 7,908 markers, 7,817 were ordered into 13 linkage groups based on the reference genome. The length of the high-density linkage map was 1833.89 cM, and the average distance between markers was 0.23 cM (**Supplementary Figure 4**; **Table 2**; **Supplementary Table 4**). The linkage group with the highest number of markers was LG5, which contained 1,667 markers. We next performed a quality assessment analysis of the genetic map. First, based on haplotype map analysis of recombination breakpoints, 7,817 markers were used to genotype the RILs, and the sources of recombination blocks were specifically explained (**Supplementary Figure 5**). Second, we analyzed the relationships between the positions of all mapped markers in the genetic map and the physical map of the reference genome, and the Spearman correlation coefficient between them exceeded 0.89, with a high observed collinearity (**Supplementary Figure 6**; **Supplementary Table 5**). Third, we used a heatmap to directly reflect recombination rates and LOD scores between markers, and no switched alleles were discovered; switched alleles were indicated by low LOD scores and low recombination fractions (**Supplementary Figure 7**) (Maldonado-Taipe et al., 2022). In summary, we constructed an accurate and reliable genetic map which was suitable for QTL mapping.

**Table 2:** Basic information of the high-density genetic linkage map of RIL population.

Linkage group ID	Total marker	Total distance (cM)	Average distance (cM)	Max gap (cM)	Gaps < 5cM (%)
LG1	333	125.16	0.38	6.20	98.50
LG2	340	155.93	0.46	13.84	97.94
LG3	691	153.39	0.22	7.04	99.42
LG4	533	146.11	0.27	9.34	98.31
LG5	1667	142.25	0.09	12.18	99.94
LG6	881	125.09	0.14	8.40	99.55
LG7	817	143.49	0.18	7.72	99.14
LG8	508	164.67	0.32	11.47	98.62
LG9	402	121.01	0.30	9.74	99.00
LG10	784	127.72	0.16	8.53	99.74
LG11	297	121.37	0.41	11.41	97.64
LG12	527	143.52	0.27	10.41	99.43
LG13	37	164.19	4.56	18.37	67.57
Total	7817	1833.89	0.23	18.37	99.08

### 3.4 BSA and QTL mapping reveal the physical position of the locus controlling the brown seed coat in sesame

**Figure 2:**
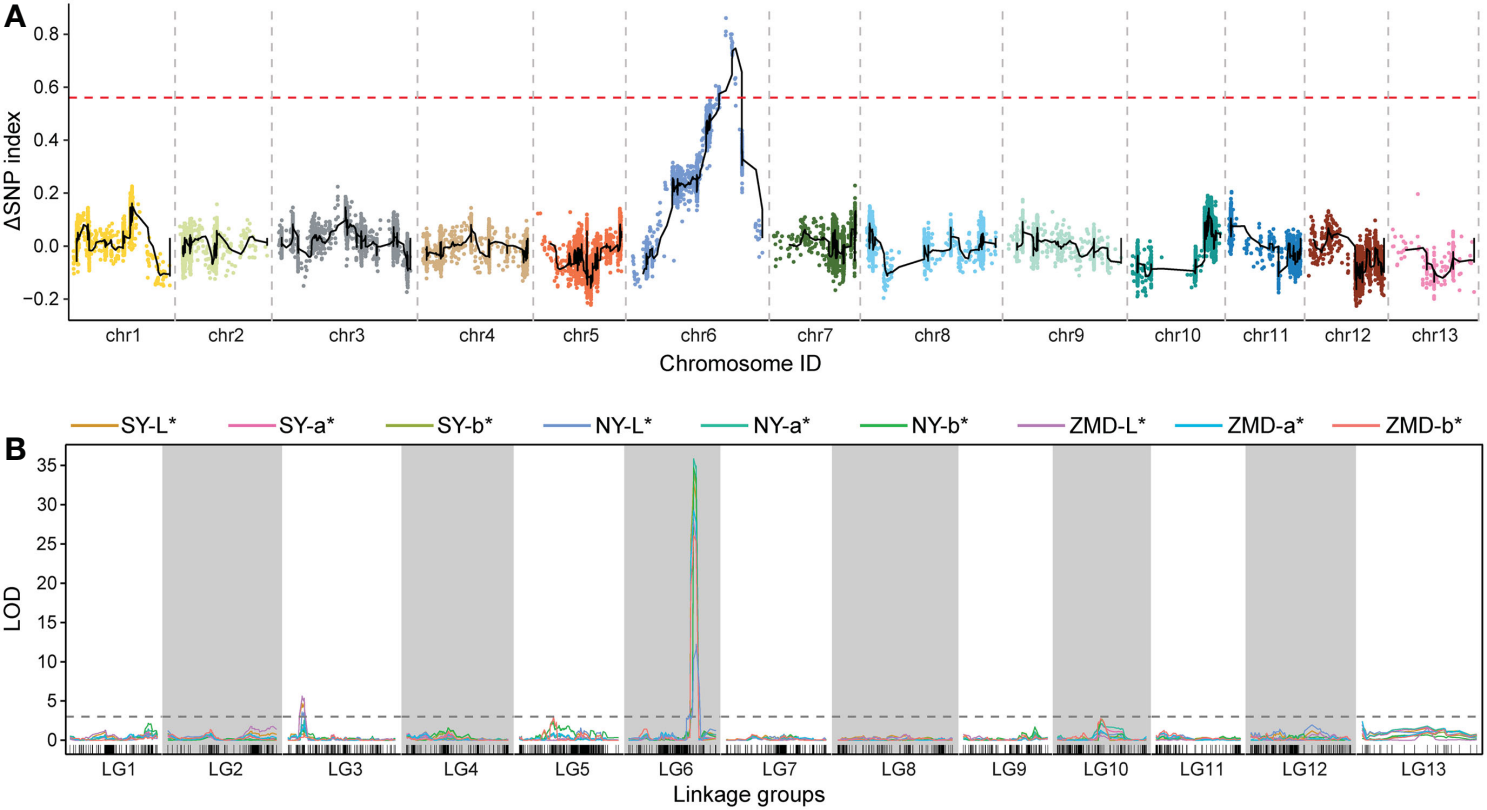
QTL identification across chromosomes and linkage groups using BSA and genetic linkage mapping, respectively. **(A)** QTL-seq analysis with the number of SNPs as the sliding window, with the red dashed line representing the significance threshold. **(B)** QTL scanning of the brown seed coat for the total linkage groups.

**Figure 3:**
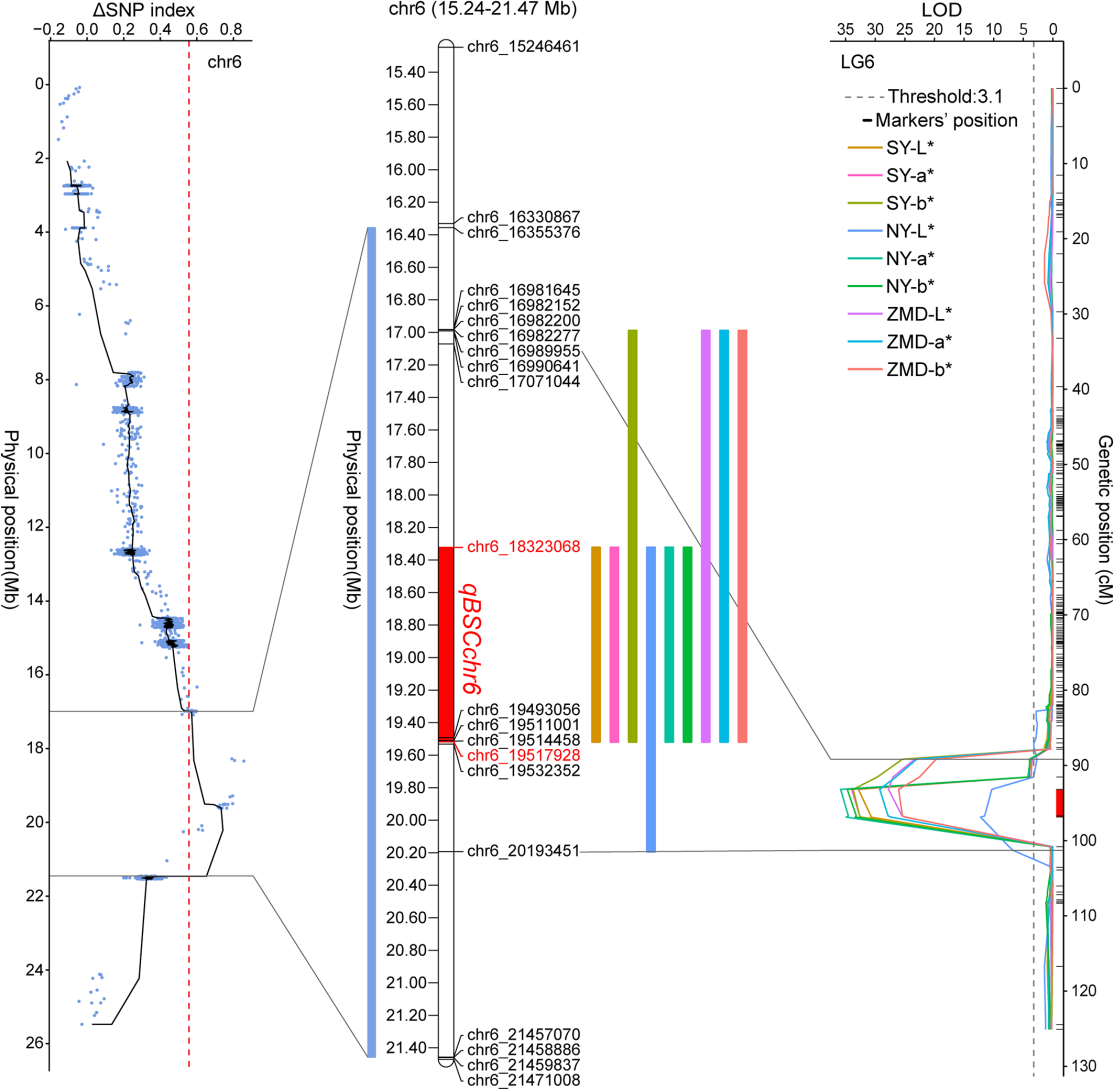
Position of *qBSCchr6* on the chr6 physical map. Colored boxes represent the physical distance spanned on chr6 for QTL identified by BSA and QTL mapping.

We identified QTLs using both BSA and traditional linkage mapping methods. In BSA, the SNP index of the two extreme bulks was calculated and visualized using sliding window analysis along chromosomes. Based on a 99.9% quantile threshold, we identified a significant physical interval (16.36 Mb~21.46 Mb) on chr6 by analyzing the SNP index of the two bulks throughout the 38,752 SNP markers (**Figure 2A**). In particular, the mean SNP index of the two bulks within the 18,323,068 to 20,213,179 bp sliding window was 0.89 and 0.14, respectively (**Supplementary Table 6**). This result suggests that there was a strong signal in this genomic region which may be controlled by a powerful QTL. To map brown seed coat-related QTLs more accurately, linkage mapping was performed based on the high-density genetic map and quantitative data for RILs seed coat color. We examined QTLs in three environments for L*, a*, and b* values. Under the threshold condition of LOD≥3.10 (*p* value=0.05), three major QTLs were detected in all three environments within a genetic interval of 89.17~101.29 cM on chr6 (**Figure 2B**; **Table 3**). The mean LOD values of the QTLs for L*, a*, and b* in the three environments were 24.27, 33.02, and 31.51, respectively, and the mean *R^2^* were 33.64%, 36.63%, and 34.55%, respectively. Additionally, a weaker QTL on chr3 for the L* value was detected in all three environments. The mean LOD value of the QTL was 4.34, and the mean *R^2^* was 4.41%, which suggests that this QTL plays a minor role in regulating brown seed coat brightness (**Table 3**). We continued our analysis of the intervals on chr6 identified by BSA and QTL mapping. Both analysis methods repeatedly identified approximately the same interval. This supports the identification of this interval and its surrounding region as a reliable major-effect QTL controlling brown seed coat traits. The flanking markers chr_16989955 and chr_20193451 spanned a physical distance of 3.2 Mb in the reference genome (chr6: 16.99 Mb~20.19 Mb). Notably, the 1.19 Mb region on chr6 between the markers chr_18323068 and chr_19517928 overlapped with other QTL intervals identified in all environments and is the closest to the LOD peak (**Figure 3**; **Table 3**). In summary, by combining BSA and traditional QTL mapping methods, we confirmed the mapping of major-effect QTL regulating the brown coat trait in sesame in the merged region of 18,323,068~19,517,928 bp on chr6, with a physical distance of 1.19 Mb. We designated this QTL *qBSCchr6*.

**Table 3:** QTL information for brown seed coat-related traits detected in the RIL population.

Trait	Environment	chr	Position (cM)	LOD	*R^2^* (%)	Start (cM)	End (cM)	Physical interval (bp)
L*	SY	3	21.60	4.46	4.31	17.00	23.29	22465300-23218480
	NY		21.60	3.42	4.05	21.60	23.11	22465436-22607606
	ZMD		21.60	5.13	4.87	17.00	23.29	22465300-23218480
	SY	6	93.19	32.84	35.68	93.19	96.87	18323068-19517928
	NY		96.87	12.21	35.79	93.19	101.29	18323068-20193451
	ZMD		93.19	27.76	29.46	89.17	96.87	16989955-19517928
a*	SY	6	93.19	33.97	37.79	93.19	96.87	18323068-19517928
	NY		93.19	35.85	39.62	93.19	96.87	18323068-19517928
	ZMD		93.19	29.25	32.49	89.17	96.87	16989955-19517928
b*	SY	6	93.19	33.74	36.02	89.17	96.87	16989955-19517928
	NY		93.19	34.73	38.60	93.19	96.87	18323068-19517928
	ZMD		93.19	26.06	29.02	89.17	96.87	16989955-19517928

### 3.5 Screening of candidate genes and preliminary validation by qRT−PCR

To extract additional information for *qBSCchr6*, we identified a total of 1,720 SNPs/InDels in this interval, among which there were 50 effective SNPs and 16 effective InDels (**Supplementary Table 7**). In total, there were 118 genes in this candidate region, with intro variants, frameshift variants, disruptive inframe deletions, and missense variants of 45, 8, 4, and 29, respectively (**Supplementary Table 8**). Ultimately, 42 genes were predicted to show high and moderate variance effects on protein coding (**Supplementary Table 9**). It was previously reported that seed coat color may be associated with the synthesis of flavonols, anthocyanins, lignin, and melanin (Pourcel et al., 2007; Yu, 2013). We found that 13 of these 118 genes may be associated with brown seed coat color regulation based on their function. Five of these genes showed high or moderate effects on protein coding; SIN_1023218, SIN_1023231, SIN_1023270, and SIN_1023287 were annotated as missense variants, and SIN_1023210 was annotated as a frameshift variant and disruptive in-frame insertion. These variants with high or moderate effects on protein coding may cause the loss of the original function and thus interrupt the accumulation of pigments in the seed coat (**Supplementary Table 10**).

**Figure 4:**
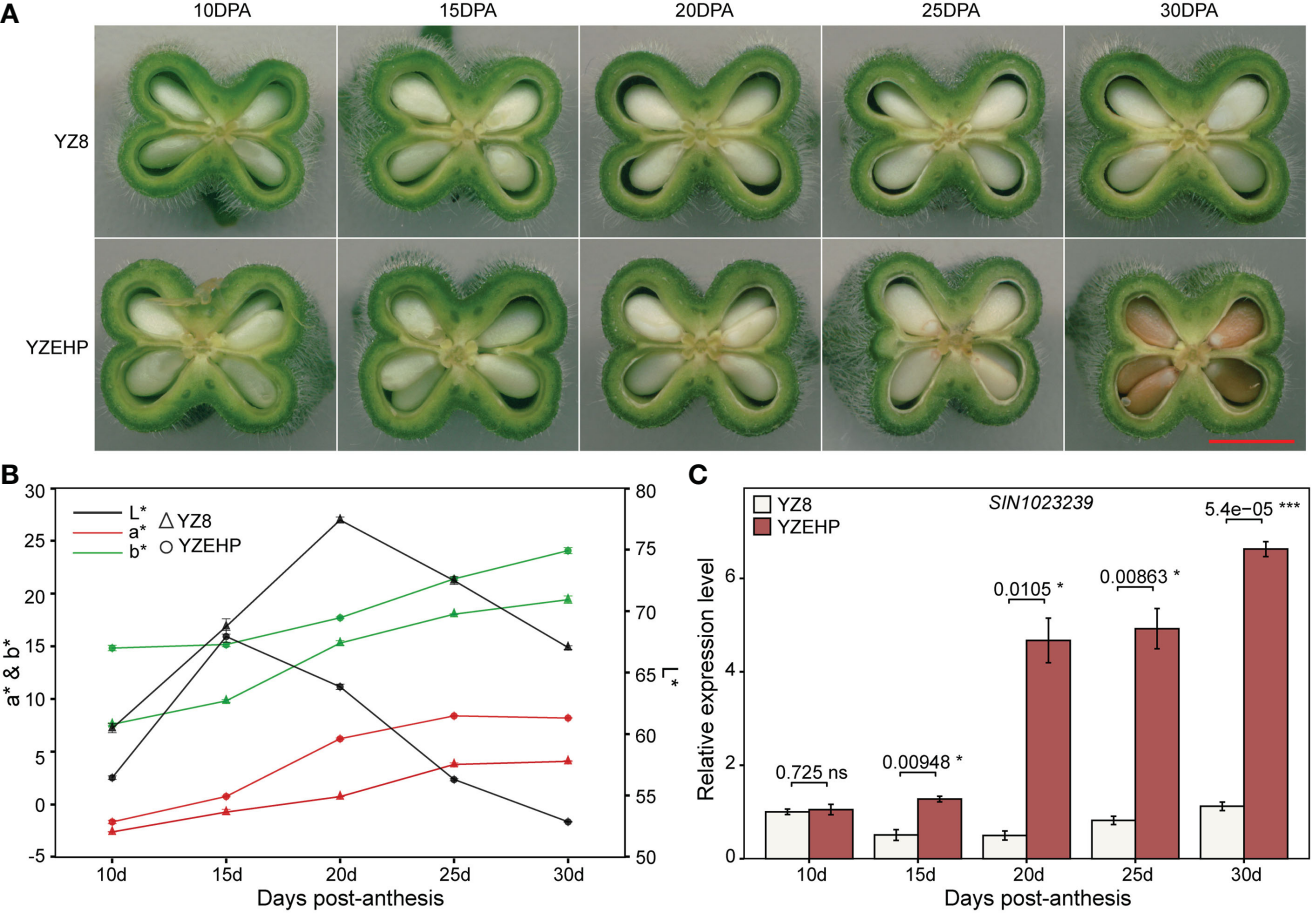
Phenotype and gene expression of parental seed coat at different developmental stages. **(A)** Longitudinal sections of capsules, red line segments indicate 0.5 mm. **(B)** Values of L*, a*, and b* for different developmental stages of the parental seed coat. **(C)** Relative expression of SIN_1023239 gene in the two parents. Significant levels of relative gene expression were determined by T-test, with ns, *, and *** representing nonsignificant, and significant at *p*<0.05 and *p*<0.001 levels, respectively.

Additionally, we observed the phenotypes of the parental characteristics at different days post-anthesis and found that the seed coat color appeared slightly different between the parents starting at 20 DPA, and that some areas of the seeds of YZEHP were colored at 25 DPA and substantially colored at 30 DPA (**Figures 4A**, **B**). Next, we performed preliminary qRT−PCR validation of 13 genes with possible functions associated with seed coat color and found that the expression level of SIN_1023239 in YZEHP was significantly up-regulated than YZ8 with 2.5-, 9.4-, 6.0-, and 5.9-fold at 15 DPA, 20 DPA, 25 DPA, and 30 DPA, respectively (**Figure 4C**). There was no discernible pattern in the expression of the remaining 12 genes in white seeds of YZ8 and brown seeds of YZEHP (**Supplementary Figure 8**; **Supplementary Table 11**). Therefore, it was the expression pattern of SIN_1023239 that was consistent with the color accumulation characteristics of the brown seed coat in YZEHP, and thus, it may be crucial for brown seed coloration.

## 4 Discussion

Seed coat color is a commercially important trait in sesame; seeds with different coat colors show specific characteristics in terms of microelement content, and it aids in the indirect selection of genotypes with high mineral content (Pandey et al., 2017). We performed separate observations and instrumental quantifications of RIL population phenotypes, and used the whole-genome resequencing technique and two computational analysis methods to map QTLs for the sesame brown seed coat trait. *qBSCchr6* was identified as a major-effect QTL that spans a physical interval of 1.19 Mb on chr6. Moreover, based on the effect of gene variants on protein coding and the potential expression pattern of the gene for pigment accumulation during seed coat development, we identified possible candidate genes within this interval.

Laurentin and Benítez (2014) developed four F_2_ populations using two white sesame cultivars and one brown sesame cultivar in reciprocal crosses, and phenotypic investigations revealed that all showed consistency with a 3:1 segregation ratio and that brown was dominant to white. This is consistent with our observation that dominant genes controlled the brown seed coat. However, the bimodal continuous distribution of L*, a*, and b* values in the RIL population indicates that a minor-effect genetic locus may also control the brown seed coat trait. Therefore, the use of high-throughput phenotypic data and an increased marker density are both effective ways to improve the efficacy of QTL detection (Li et al., 2010). In addition, the values of L*, a*, and b* obtained in the three environments, showed high heritability. Previous studies have also demonstrated that over 90% of the phenotypic variation in sesame seed coat color is genetically controlled and slightly influenced by environmental factors (Zhang et al., 2013). Moreover, due to indeterminate inflorescence growth, climate, and harvest time, differences in seed maturity at harvest can also cause differences in seed coat color, leading to instability in phenotypic and QTL analyses, as reported based on seed coat color mapping in *Brassica napus* (Yan et al., 2009). Interestingly, in the present study, the mean CV (from high to low) were 27.86%, 12.80%, and 6.54% for a*, b*, and L* values, respectively, indicating that a* value had the highest dispersion in the RIL population and best represented the phenotypic characteristics of the brown coat color trait in sesame, while the opposite was true for L* value.

A high-quality genetic map is the basis of QTL mapping for agronomic traits. QTL mapping by whole-genome low coverage sequencing has been successfully applied to chickpea and peanut (Kale et al., 2015; Sun et al., 2022). In these studies, the parental sequencing depths ranged from ~7.9× to 34.58×, the population sequencing depths ranged from 0.72× to 1.4×, and the number of markers used for mapping ranged from ~53,000 to ~210,000. The actual sequencing coverage obtained in whole-genome resequencing averaged 18.61× in the parents and 5.16× in the RIL population, which was considered sufficient for QTL mapping in this study (**Supplementary Table 2**). Although the number of markers we obtained for mapping was only ~160,000, possibly due to our strict filtering of the marker sequencing depth, this did not affect our ability to construct a reliable and stable genetic map and use it for subsequent QTL mapping. In addition, we found that most of the linkage groups were separated into subgroups due to the uneven distribution of adjacent markers and large gaps (up to ~18 cM), and the calculation of recombination scores was affected by the lack of markers. We further validated collinearity with physical maps (such as LG8, LG10, and LG12) and found that most markers were located in the central region of chromosomes, allowing each chromosome to be split into several contiguous groups, similar to what has been found in wheat and quinoa (Langlands-Perry et al., 2021; Maldonado-Taipe et al., 2022). Importantly, this did not affect our subsequent QTL mapping analysis, which passed several independent tests for quality.

Most previous studies on QTLs regulating sesame seed coat color have included co-mapping for black sesame or segregation of various colors and have not been able to separate the QTLs or mechanisms of interaction mapped to individual seed coat colors. Through 10 successive generations of self-fertilization, we created a population of RILs with stable inheritance and eventually identified a major-effect QTL controlling brown seed coat traits on chr6. Furthermore, we compared *qBSCchr6* with QTLs associated with seed color from previous reports. However, only the results from a genome-wide association study (GWAS) of seed coat color in 366 natural populations included the same physical interval (Cui et al., 2021). In particular, most of the significant SNPs in the GWAS results were mapped to the confidence intervals of *qSCa-4.1*/*qSCb-4.1*/*qSCl-4.1*, *qSCa-8.1*/*qSCb-8.1*/*qSCl-8.1* and *qSCl-8.2* identified by Wang et al. (2016), which further suggests the specificity and accuracy of *qBSCchr6* in controlling brown seed coat color. Other comparable QTLs were not mapped to our confidence interval (Wei et al., 2015; Wang et al., 2016). Some previous studies applied independent genetic maps and genomes, making it difficult to determine the relationships between their results and *qBSCchr6* (Zhang et al., 2013; Du et al., 2019; Li et al., 2021). In the present study, the linkage analysis also revealed a minor-effect QTL for L* color values on chr3 across the three environments, with LOD values between 3.42 and 5.13 and *R^2^* between 4.05% and 4.87% (**Table 3**). However, the ΔSNP index in the BSA did not fluctuate within this interval, probably because QTL-seq is not suitable for detecting minor-effect QTLs without the repeated measurement of phenotypes across multiple years (Takagi et al., 2013). Phenotypic data also showed the smallest dispersion of L* values in the three environments, and it is possible that weak changes in brightness do not cause visually detectable differences.

The presentation of seed color in various plants is complex and diverse, involving the main components of flavonols, PAs (concentrated tannins), and some phenolic substances such as lignins and melanins (Yu, 2013). We sampled seeds every 5 days from 10 DPA until we observed significant differences in the seed coat color between the parental plants. From 20 DPA onward, we observed the greatest variation in L* values, with YZEHP seeds being darker than YZ8 seeds, and we eventually noted a clear color difference at 30 DPA. Wang et al. (2020) found that black sesame seeds started to synthesize and accumulate melanin gradually at 8 DPA and that a significant difference in seed coat color appeared at 14 DPA. These results were not exactly the same as ours, and we speculate that this might be due to the different metabolic pathways involved in the accumulation of pigmented substances. A search for candidate genes within the confidence interval of *qBSCchr6* was further performed. Among the 13 screened genes, SIN_1023210 has been annotated as encoding the UDP-glycosyltransferase 87A2 protein associated with catalytic glycosylation (one of the final steps in the production of secondary metabolites) and plays an important role in determining the coloration of flowers, leaves, seeds, and fruits (Le Roy et al., 2016; Foong et al., 2020). SIN_1023231 and SIN_1023270 are annotated as exocyst subcomplex-containing subunit (EXO70) proteins associated with the vesicle-dependent autophagy-related pathway of anthocyanin-containing vesicles from the endoplasmic reticulum into the vesicle lumen (Kulich et al., 2013). SIN_1023248, SIN_1023249, SIN_1023303, and SIN_1023305 all encode peroxidases, which may be related to lignin formation and coloration during fruit ripening (Pourcel et al., 2007; Ring et al., 2013). SIN_1023218 encodes alanine glyoxylate aminotransferase 2, which is involved in the transfer and catalysis of amino acids (Liepman and Olsen, 2003). SIN_1023221 and SIN_1023287 encode 2-oxoglutarate-dependent dioxygenase and beta-glucosidase, respectively, which are essential enzymes in flavonoid and phenylpropanoid biosynthesis (Farrow and Facchini, 2014; Munir et al., 2019). These are all potential regulatory pathways related to seed coat pigment accumulation. Furthermore, SIN_1023237, SIN_1023239, and SIN_1023240 all encode laccase 3 (LAC3), a multicopper glycoprotein that catalyzes and activates the oxidation of diphenol substrates in the presence of molecular oxygen in poplar (Ranocha et al., 1999). However, we found that only SIN_1023239 was significantly up-regulated in YZEHP seeds at different developmental periods compared to its expression in YZ8, and the expression pattern was consistent with the phenotypic trend. In *Arabidopsis*, *TT10* (laccase 15) is involved in the oxidation of concentrated tannins in the seed coat, resulting in brown coat color at harvest, and the other 16 laccase enzymes do not seem to compensate for the loss of activity in the TT10 mutant (Pourcel et al., 2005). In addition, preliminary evidence based on bioinformatics suggests the presence of one or more forms of epigenetic modification in the coding sequences of the eight laccase enzymes including AtLAC3 (Turlapati et al., 2011). In poplar, *LAC3* increased the content of soluble phenols in the seed coat, participated in the oxidation of lignin, and affected the structure and integrity of the cell wall (Ranocha et al., 2002). In maize, *ZmLAC3* is also involved in the polymerization of phenolic compounds (Caparrós-Ruiz et al., 2006). In addition to flavonoids and anthocyanins, some researchers have surmised that lignins or phenolics affect the seed colors of plants, although the available evidence is not sufficient to support this conclusion (Qu et al., 2013). A recent study by Dossou et al. (2022) focused on the metabolomics of four sesame cultivars and found that the developmental regulation of black, brown, yellow, and white sesame seed coat colors may be different, resulting in different coloration due to variations in the major bioactive phenolic compounds in sesame seeds. Nevertheless, our identification of long fragments of InDels or SNPs may be missed. Further development of markers for fine mapping is needed, and multiomics techniques should be combined to analyze the deposition of sesame seed coat pigments to identify the regulatory mechanisms underlying different color traits.

## Data availability statement

The datasets presented in this study can be found in online repositories. The names of the repository/repositories and accession number(s) can be found below: NCBI–PRJNA934094, SRR23434336 to SRR23434652.

## Author contributions

ZW, HM, and BJ directed the project and advised on subsequent studies. HW and CC designed the experiments, performed bioinformatics analysis, completed the construction of the genetic map and computational analysis of genotype frequencies, and completed gene screening and quantitative analysis. YL and YZZ developed the RIL population and performed field experiments. YQZ, XC, and XW performed the DNA extraction. HW together with all authors wrote and finalized the manuscript. All authors contributed to the article and approved the submitted version.

## Supplementary material

Supplementary Figure 1 Frequency distribution of L*, a*, and b* values in the three environments of the RIL population.

Supplementary Figure 2 Statistical information on individual sequencing data of parents and RILs. **(A)** Clean data size distribution. **(B)** Information on the mapped ratio.

Supplementary Figure 3 Statistical information on genetic markers used to construct genetic maps. **(A)** Marker type and quantity statistics. **(B)** Statistical information of valid SNP/InDel genetic markers in each linkage group.

Supplementary Figure 4 High-density genetic map of RIL population. Each vertical line represents the position of the marker in the linkage groups.

Supplementary Figure 5 Haplotype assessment of recombination breakpoints for each sample of the RIL population.

Supplementary Figure 6 Analysis of collinearity between genetic and physical maps of sesame. Horizontal coordinates indicate the genetic distance of each linkage group, and vertical coordinates indicate the physical length of each chromosome, and marker collinearity in genomic and genetic maps is represented in the form of scatter.

Supplementary Figure 7 Heatmap of pairwise recombination and LOD scores based on 7,817 markers. Estimated recombination scores between markers are shown above the diagonal line, and LOD scores are shown below the diagonal line. Red indicates closely linked markers (high LOD scores and low recombination scores) and blue indicates non-linked markers (low LOD scores and high recombination scores).

Supplementary Figure 8 Relative expression levels of candidate genes that were inconsistent with the pattern of phenotypic variation among parents at different developmental stages of the seed coat. Significant levels of relative gene expression differences between parents at each period of seed development were tested by T-test, with ns, *, **, and *** representing nonsignificant, significant at the *p*<0.05, *p*<0.01, and *p*<0.001 level, respectively.
