# Supplementary figures and images for: Genetic mapping of QTLs controlling brown seed coat traits by genome resequencing in sesame (Sesamum indicum L.)
Source: Front Plant Sci. 2023 Feb 23;14:1131975. doi: 10.3389/fpls.2023.1131975 (PMC9995652; doi:10.3389/fpls.2023.1131975)

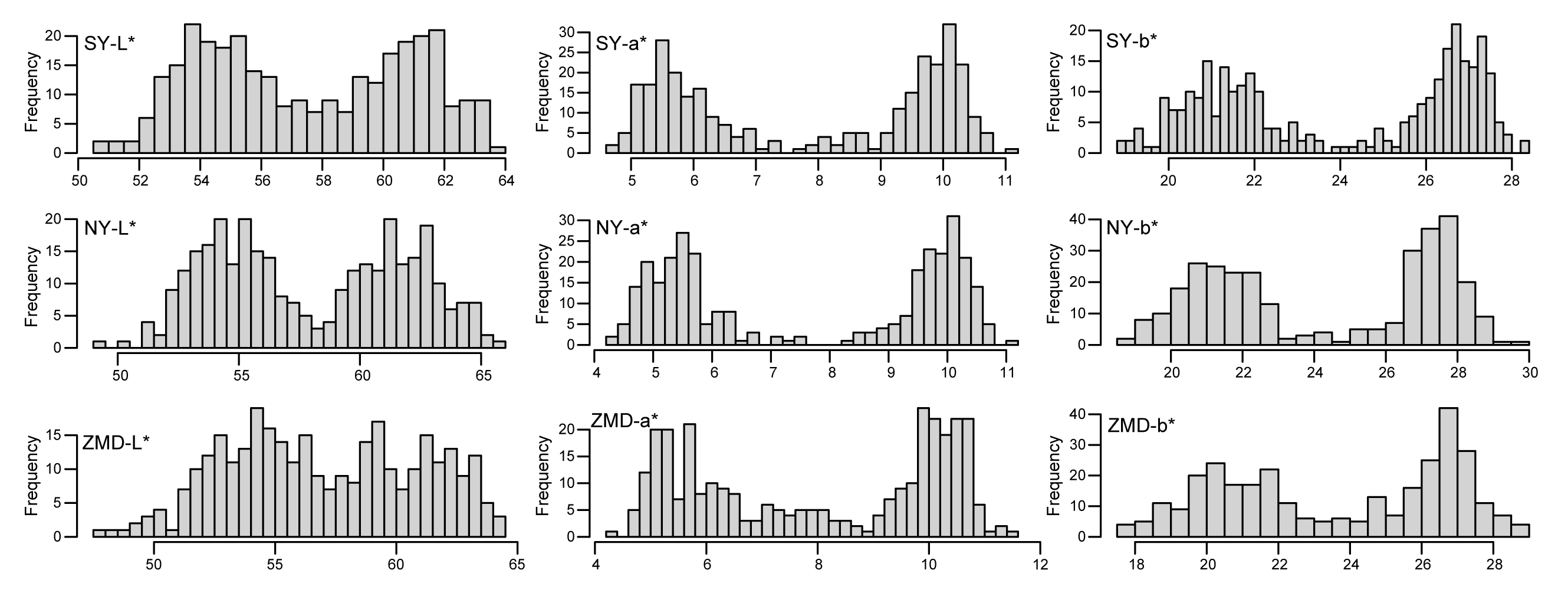

Supplement: Supplementary Figure 1 — Frequency distribution of L*, a*, and b* values in the three environments of the RIL population. [file Image_1.tif]

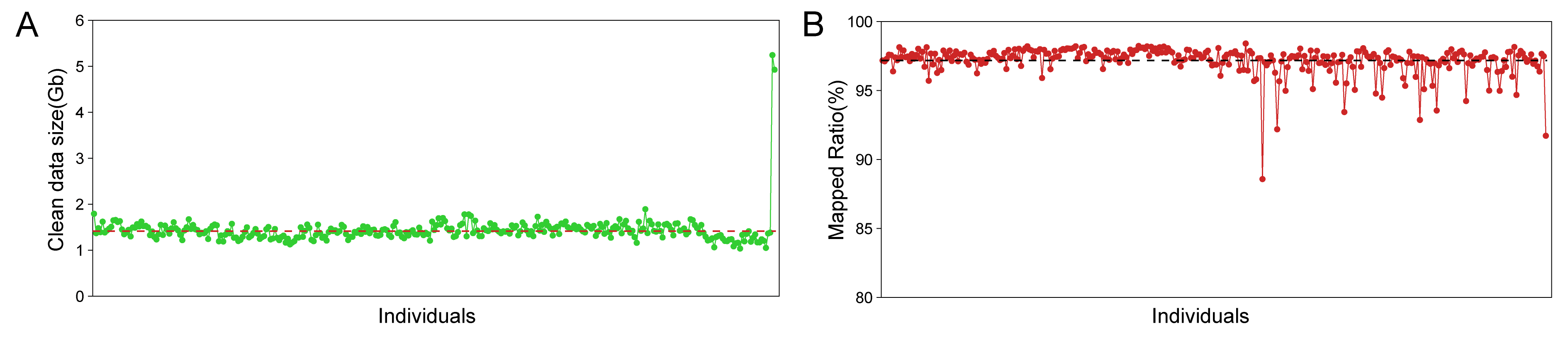

Supplement: Supplementary Figure 2 — Statistical information on individual sequencing data of parents and RILs. (A) Clean data size distribution. (B) Information on the mapped ratio. [file Image_2.tif]

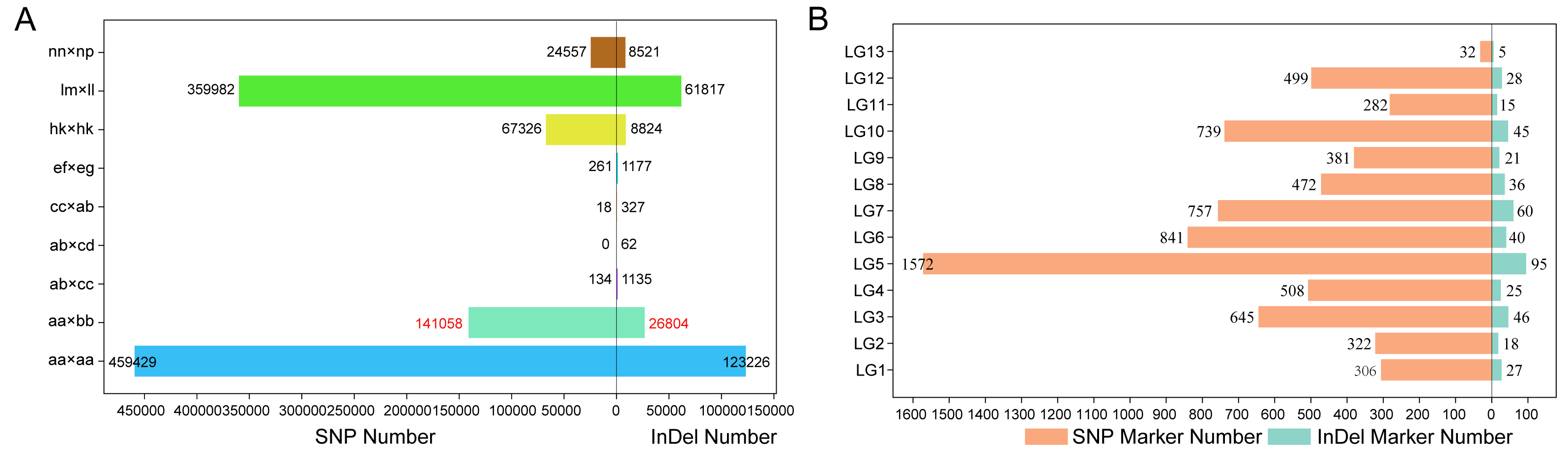

Supplement: Supplementary Figure 3 — Statistical information on genetic markers used to construct genetic maps. (A) Marker type and quantity statistics. (B) Statistical information of valid SNP/InDel genetic markers in each linkage group. [file Image_3.tif]

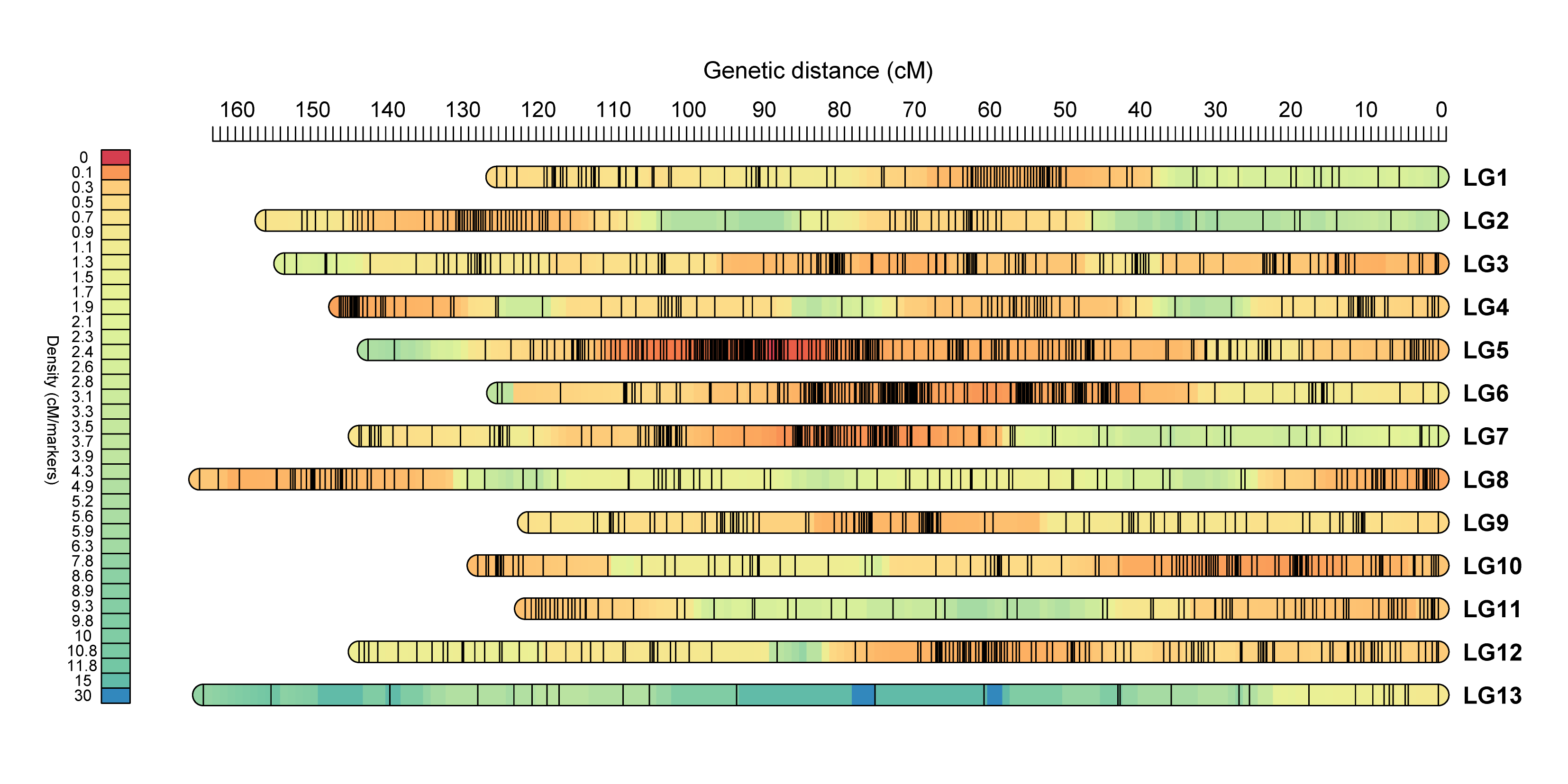

Supplement: Supplementary Figure 4 — High-density genetic map of RIL population. Each vertical line represents the position of the marker in the linkage groups. [file Image_4.tif]

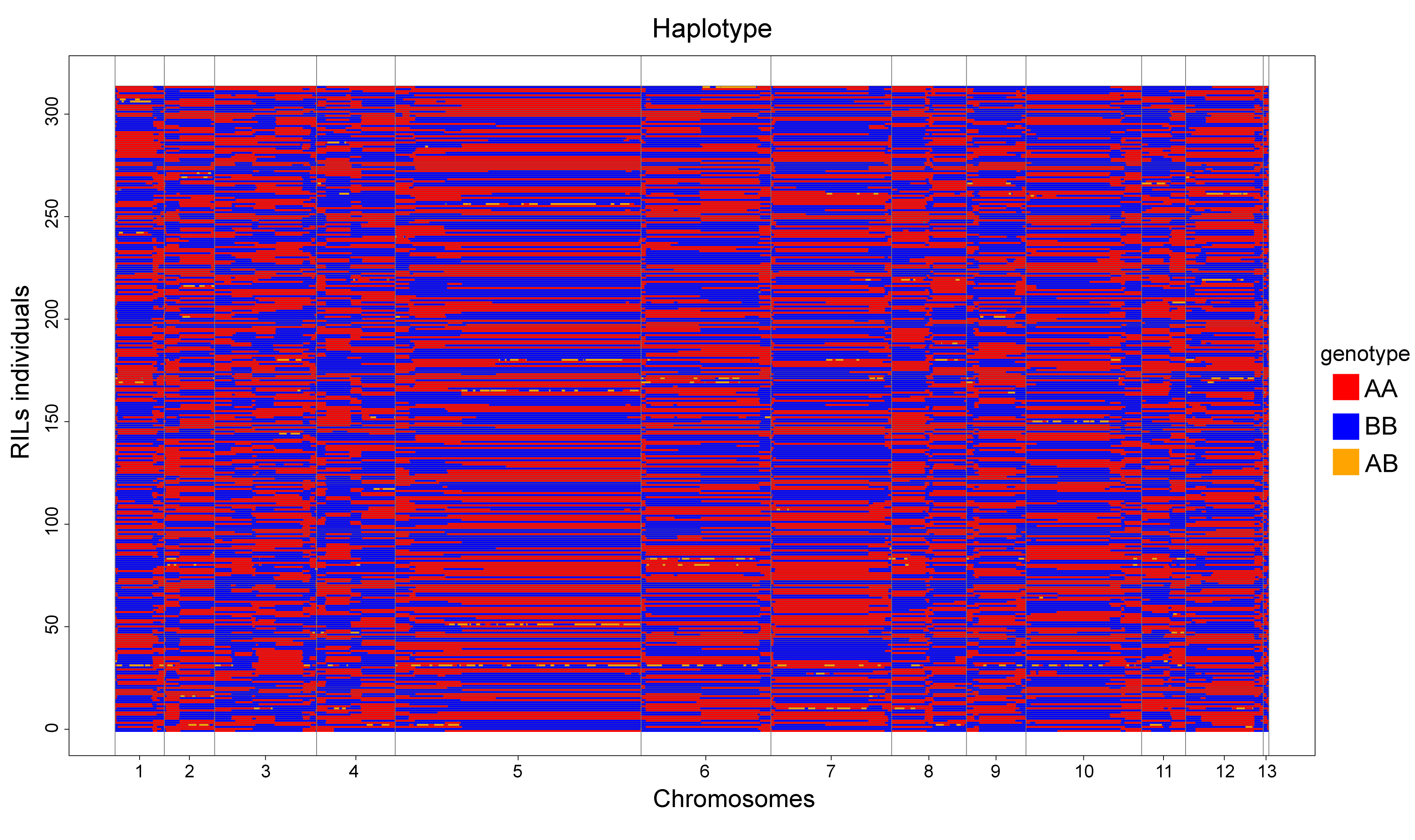

Supplement: Supplementary Figure 5 — Haplotype assessment of recombination breakpoints for each sample of the RIL population. [file Image_5.tif]

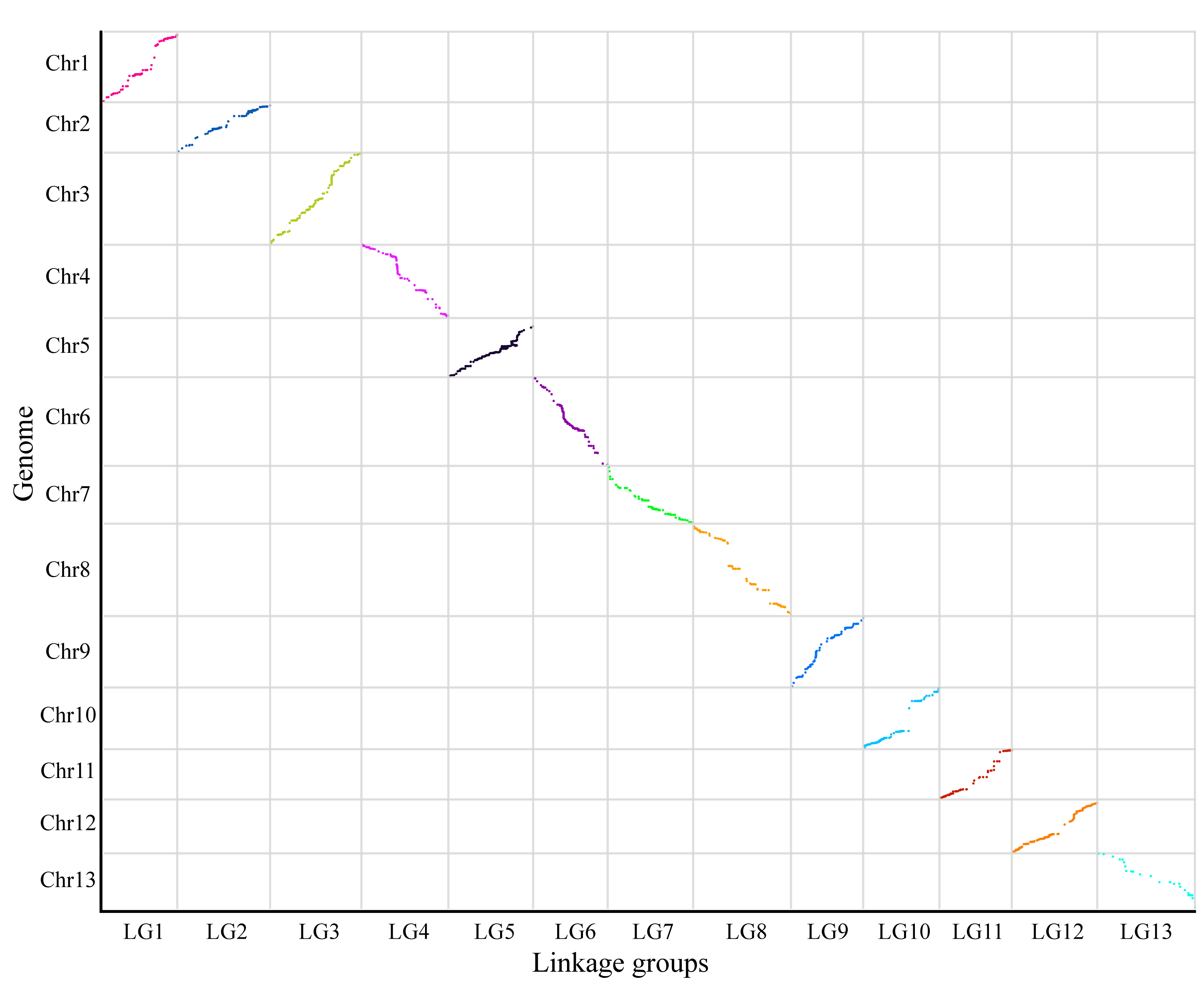

Supplement: Supplementary Figure 6 — Analysis of collinearity between genetic and physical maps of sesame. Horizontal coordinates indicate the genetic distance of each linkage group, and vertical coordinates indicate the physical length of each chromosome, and marker collinearity in genomic and genetic maps is represented in the form of scatter. [file Image_6.tif]

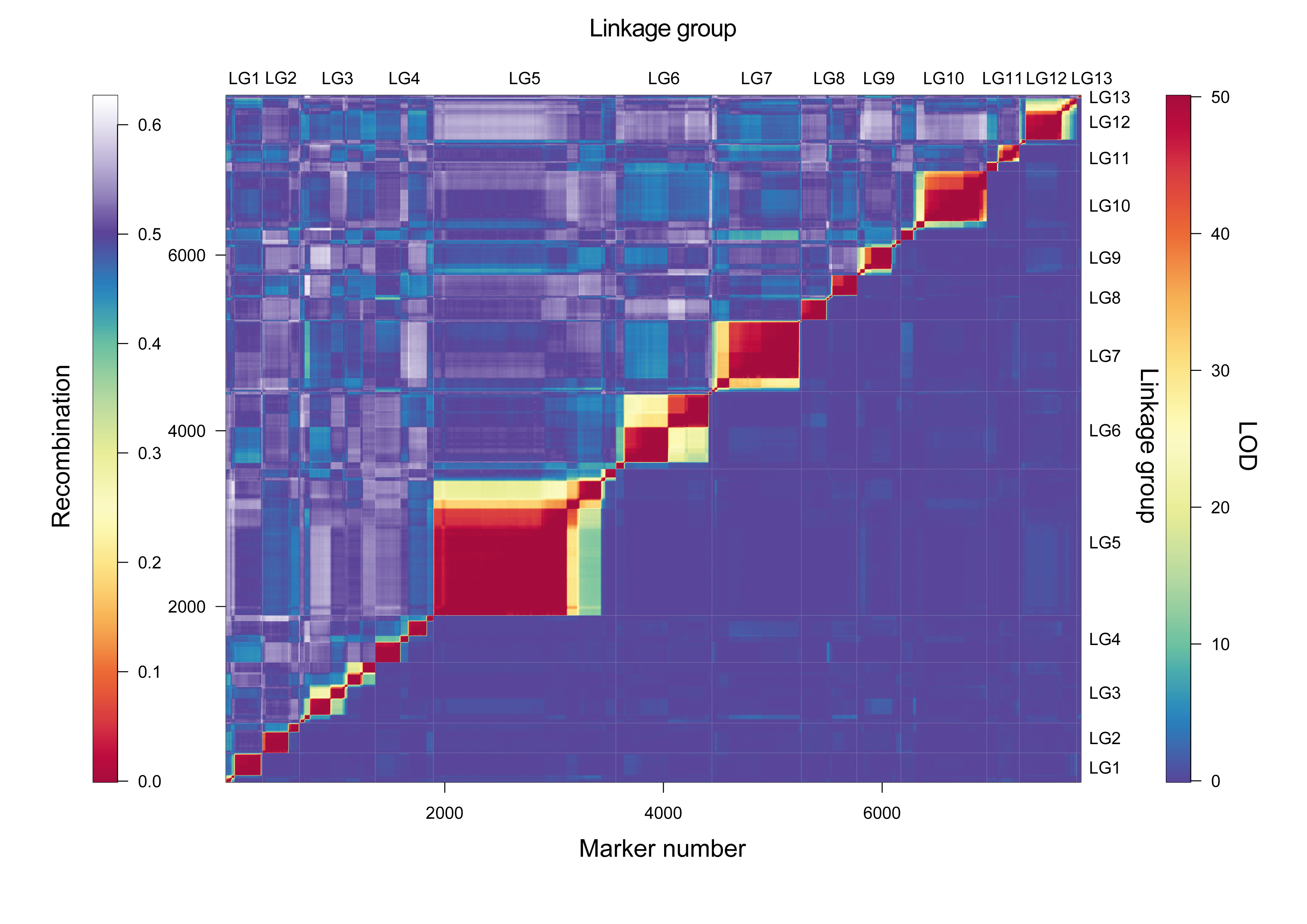

Supplement: Supplementary Figure 7 — Heatmap of pairwise recombination and LOD scores based on 7,817 markers. Estimated recombination scores between markers are shown above the diagonal line, and LOD scores are shown below the diagonal line. Red indicates closely linked markers (high LOD scores and low recombination scores) and blue indicates non-linked markers (low LOD scores and high recombination scores). [file Image_7.tif]

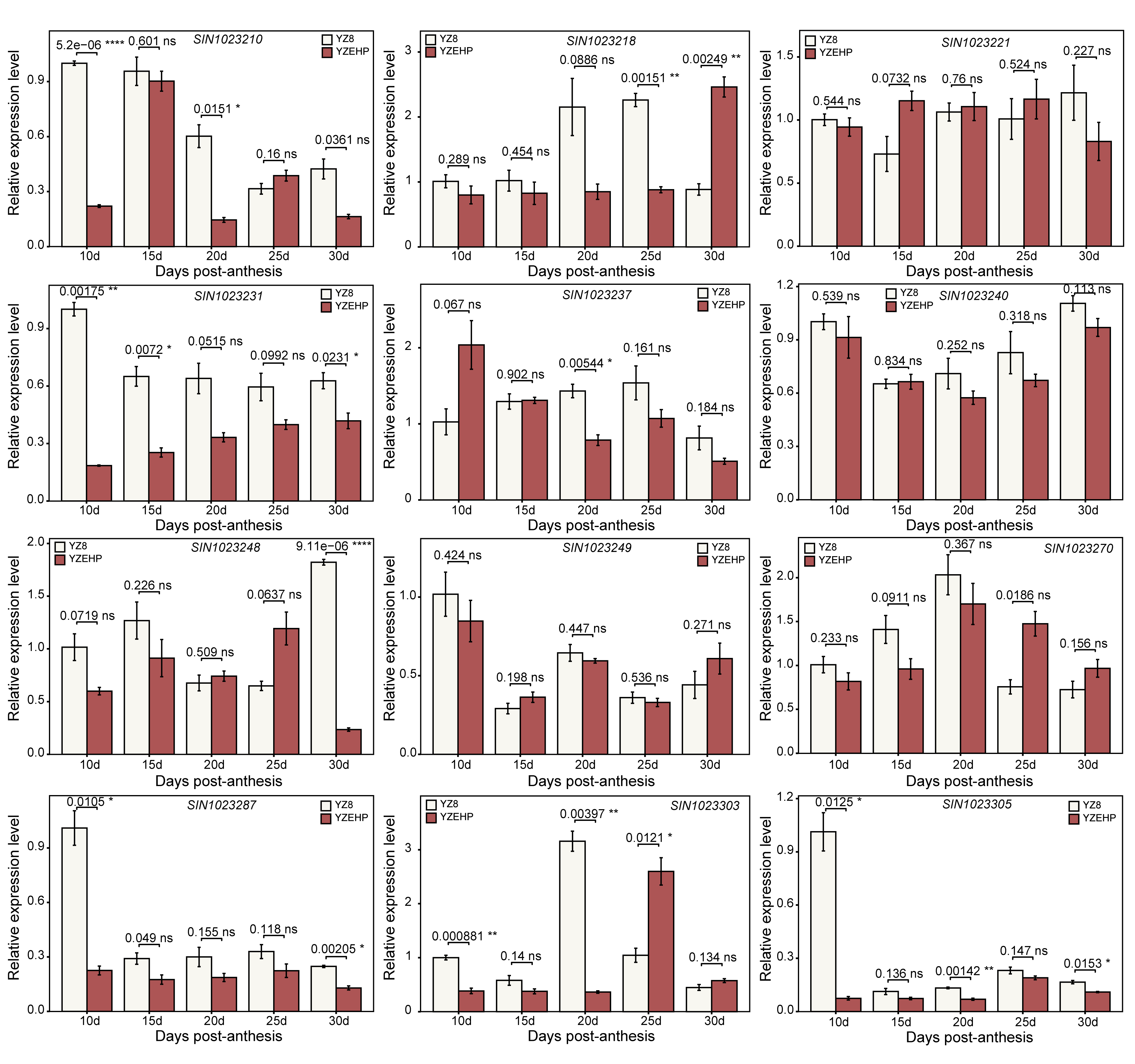

Supplement: Supplementary Figure 8 — Relative expression levels of candidate genes that were inconsistent with the pattern of phenotypic variation among parents at different developmental stages of the seed coat. Significant levels of relative gene expression differences between parents at each period of seed development were tested by T-test, with ns, *, **, and *** representing nonsignificant, significant at the p<0.05, p<0.01, and p<0.001 level, respectively. [file Image_8.tif]
